# New species of *Lathrolestes* Förster (Hymenoptera: Ichneumonidae) from Côte d’Ivoire

**DOI:** 10.3897/BDJ.1.e1005

**Published:** 2013-12-10

**Authors:** Alexey Reshchikov

**Affiliations:** † Department of Zoology, Swedish Museum of Natural History, Stockholm, Sweden

**Keywords:** Ctenopelmatinae, Perilissini, *
Lathrolestes
*, *Lathrolestes ivoriensis*, *Lathrolestes ruwenzoricus*, Afrotropical region, Côte d’Ivoire.

## Abstract

*Lathrolestes ivoriensis* **sp. n.** is described from Côte d’Ivoire. This is the second record of the genus from the Afrotropical region and the first record of the genus and the subfamily for the country. Illustrated re-description of *Lathrolestes ruwenzoricus* (Benoit, 1955) is also provided.

## Introduction

Very few is known about the diversity of Afrotropical Ichneumonidae, excluding the excellent revision of Ophioninae by Gauld & Mitchell (Gauld and Mitchell 1978), seminal work by Seyrig (Seyrig 1935) and a series of recent papers (Bennett and Barnes 2011, Rousse et al. 2013, Yu et al. 2012). However most of those paper were focused in Madagascar or South Africa. Only 1979 species of ichneumonids had been recorded from the whole Afrotropical region (Yu et al. 2012). This is less than half as many as are known from Germany! The African fauna of the large subfamily Ctenopelmatinae (Hymenoptera, Ichneumonidae) is very poorly known – there are currently only ten species known from tropical Africa (Aubert 1987, Benoit 1955, Cameron 1911, Morley 1926, Seyrig 1935, Szépligeti 1908, Townes and Townes 1973).

The genus *Lathrolestes* Förster, 1869 (Hymenoptera, Ichneumonidae) is a large genus with 99 described species (Reshchikov et al. 2012, Reshchikov 2013). Only one species was previously described from the Afrotropical region (Benoit 1955). No species of *Lathrolestes* had been recorded in Côte d’Ivoire or anywhere else in Africa except Democratic republic of the Congo (Benoit 1955) before this work. One species, *Lathrolestes ivoriensis* sp. n. is described in this paper as new. Taking into consideration how rare species of the genus *Lathrolestes* are in Africa, and the distinguishing combination of character states, this species is described from only one specimen.

## Materials and methods

This work is based on the material of the Royal Museum for Central Africa (RMCA), Tervuren, Belgium. Morphological terminology used in the study follows that of Gauld (Gauld 1997). The female holotype specimen of *Lathrolestes ruwenzoricus* (Benoit, 1955) was examined, re-described and illustrated. Photographs were taken with a Canon Digital Camera 7D, combined with Zerene®. The following collections were checked for Afrotropical material: American Entomological Institute, Gainesville; Academy of Natural Sciences of Philadelphia; Hungarian Museum of Natural History, Budapest; Natural History Museum, London; National Museum of Natural History, Paris; "Naturalis" Biodiversity Centre, Leiden; Swedish Museum of Natural History, Stockholm; University of Tartu. Not any specimens of the genus *Lathrolest* were found in these collections.

## Taxon treatments

### Lathrolestes ivoriensis

Reshchikov sp. n.

urn:lsid:zoobank.org:act:B7B1AEA3-2DDD-48DE-B9AF-71384AFCFE5A

#### Materials

- **Type status:** Holotype. **Occurrence:** recordedBy: J. Decelle; individualCount: 1; sex: female; **Location:** country: Côte d’Ivoire; stateProvince: Abengourou; verbatimLocality: Amangouakro; verbatimLatitude: 6°52'3.79"N; verbatimLongitude: 3°45'48.97"W; **Event:** eventDate: xii.1962; **Record Level:** institutionCode: RMCA

#### Description

Body length 10 mm (Fig. 1a). Antenna with 35 flagellomeres. Scape 1.54 times as long as wide (Fig. 1b). Head narrowed behind eyes (Fig. 2b), matt, sparsely and shallowly punctate on granulated surface. Maximum length of temple 0.63X transverse eye diameter; minimum length of temple 0.53X transverse eye diameter. Width of face 1.18X height of eye (Fig. 1b), in dorsolateral profile very slightly convex, with bulge, sparsely (more densely in the middle) and shallowly punctate on granulated surface; frons the same; interspace between hind half of lateral ocellus and eye and vertex matt, with shallow sparse punctures, 3.36X of transverse ocellus diameter (Fig. 2b). Clypeus not separated from face, sparsely punctate, very slightly projecting anteriorly (Fig. 1b); apical margin of clypeus obtuse. Tentorial pit large and elongate. Malar space 0.76X basal mandible width, its margin banded by rugosity starting from tentorial pit. Lower mandible tooth longer than upper. Occipital carina medially complete, reaching hypostomal carina at base of mandible.

Mesosoma matt. Notauli vestigial (Fig. 2b). Mesopleuron matt, granulated, with weak sparse punctures. Tarsus with apical article not enlarged (Fig. 2a). Claws pectinate till its half with not high teeth (Fig. 2a). Wings infuscate (Fig. 1a). Fore wing with areolet petiolate. Rs intercepting pterostigma before its middle. 2m-cu straight, with single bulla. Hind wing with cu-a intercepted below middle. Metapleurum granulated. Propodeum matt, impunctate, basal transverse carina and costula absent, area superomedia elongate (Fig. 2d). Body with fine moderately dense setae.

Metasoma matt, evenly covered with moderately short dense setae, shallowly punctate. First metasomal tergite 2.6X as long as apically wide; without shallow median longitudinal impression; bordered by lateral longitudinal carinae and dorsal longitudinal carinae (rather good defined in middle (Fig. 2e). Second metasomal tergite slightly elongate (Fig. 2f). Ovipositor straight, stout dorsally, without notch (Fig. 2c), as long as metasomal height.

Coloration (Figs 1, 2). Face, basal part of clypeus, malar space, basal part of mandible, temples around eye yellow. Apical part of clypeus, mandible, frons, vertex and outer part of temple black. Antenna, palps,mostly pronotum, notum (excluding horseshoe spot in its hind part), tegula, veins and pterostigma, coxae, trochanters, tarsi, hind legs entirely, hind part of 1st and 2nd metasomal tergites and entirely further tergites, ovipositor sheath dark brown. Lower part of pronotum, horseshoe spot in hind part of notum, scutellum, postscutellum, propodeum, mesopleurum, femur and tibia of fore and middle legs, 1st and 2nd (excluding their hind parts) reddish.

#### Diagnosis

This species differs from other species of the genus by the combination of the following character states: head narrowed behind eyes, clypeus not separated from face, margin of malar space banded by rugosity starting from tentorial pit, wings infuscate, claws pectinate till its half with not high teeth (Fig. 2a), basal transverse carina and costula absent, area superomedia elongate (Fig. 2d), ovipositor straight, stout dorsally, without notch (Fig. 2c), as long as metasomal height. This species morphologically closely related to *Lathrolestes jennyae* Gauld, 1997, *Lathrolestes xochiquetzalis* Reshchikov, 2011 and *Lathrolestes kukulcanis* Reshchikov, 2011 (all from Central America) with which it shares similar coloration of clypeus (yellow and black in apical part) and well defined dorsal longitudinal carinae of 1st metasomal tergite. The new species clearly differs from Mexican species by the longer first metasomal tergite (2.6X as long as apically wide versus shorter than 1.6–1.9 X as long in the Mexican species), absence of basal transverse carina of the propodeum and costula (Fig. 2d), ovipositor as long as metasomal height, straight, stout dorsally, without notch (Fig. 2c) and unique coloration (Figs 1, 2) (see description). From the single known Afrotropical species, *Lathrolestes ruwenzoricus* (Benoit, 1955) the new species is distinguished on the basis of colour (*Lathrolestes ruwenzoricus* is entirely black except for reddish fore legs).

#### Etymology

The species epithet refers to the name of the country where it was collected.

#### Distribution

Côte d’Ivoire.

### Lathrolestes ruwenzoricus

(Benoit, 1955)

#### Description

**Figure 3.**
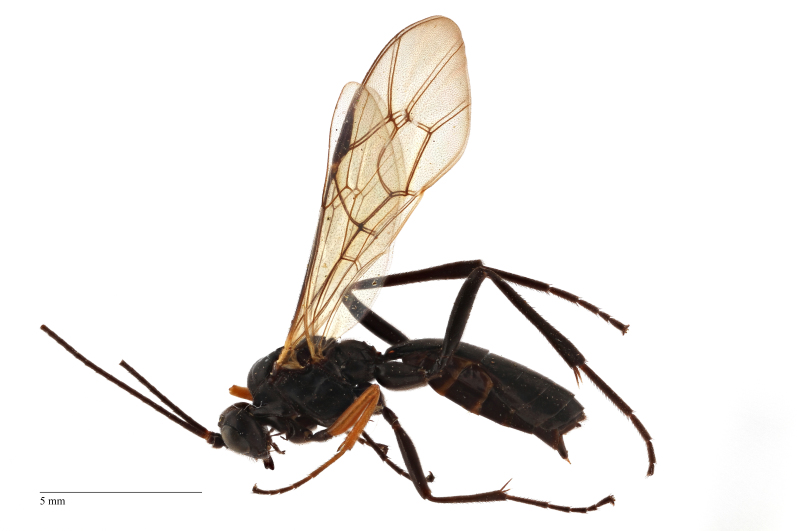
Holotype female of *Lathrolestes ruwenzoricus* (Benoit, 1955), habitus.

Body length 12 mm (Fig. 3). Scape 1.08 times as long as wide (Fig. 4a). Head narrowed behind eyes (Fig. 4b), matt, sparsely and shallowly punctate on smooth surface. Maximum length of temple 1.3X transverse eye diameter; minimum length of temple 1.07X transverse eye diameter. Width of face 1.18X height of eye; in dorsolateral profile very slightly convex, with bulge, sparsely and shallowly punctate on smooth surface; frons the same; interspace between hind half of lateral ocellus and eye and vertex matt, with shallow sparse punctures, 2.58X of transverse ocellus diameter. Clypeus not separated from face, sparsely punctate, very slightly projecting anteriorly; apical margin of clypeus obtuse. Tentorial pit large and elongate. Malar space equal basal mandible width, its margin banded by rugosity starting from tentorial pit. Lower mandible tooth longer than upper. Occipital carina medially complete, reaching hypostomal carina at base of mandible.

Mesosoma matt. Notauli shalowely impredded at base. Mesopleuron matt, smooth, with weak sparse punctures. Tarsus with apical article not enlarged (Fig. 4c). Claws pectinate (Fig. 4c). Wings hyaline (Fig. 3). Fore wing with areolet petiolate. Rs intercepting pterostigma far before its middle. 2m-cu straight, with single bulla. Hind wing with cu-a intercepted below middle. Metapleurum slightly wrinkled. Propodeum matt, impunctate, only apical areas defind (Fig. 4d). Body with fine moderately dense setae.

Metasoma matt, evenly covered with moderately short dense setae, shallowly punctate. First metasomal tergite 1.52X as long as apically wide (Fig. 4e); witho very weak shallow median longitudinal impression; bordered by lateral longitudinal carinae, dorsal longitudinal carinae absent. Second metasomal tergite square (Fig. 4f). Ovipositor straight, without notch, as long as metasomal height.

Coloration (Figs 3, 4). Body mostly black. Fore femur, tibia and basal tarsomer reddish.

#### Diagnosis

This species differs from other members of the genus by the combination of the following character states: body mostly black excluding fore femur, tibia and basal tarsomerus which are reddish; matt, sparsely and shallowly punctate on smooth surface; Rs intercepting pterostigma far before its middle; propodeum matt and impunctate with only apical areas defind. This species very easily can be recognized from *Lathrolestes ivoriensis* Reshchikov, sp. n. by unusually black and dim coloration of body, propodeal carinae with only apical areas present and absence of dorsal longitudinal carinae of 1st metasomal tergite.

#### Distribution

Democratic Republic of the Congo.

## Supplementary Material

XML Treatment for Lathrolestes ivoriensis

XML Treatment for Lathrolestes ruwenzoricus

## Figures and Tables

**Figure 1a.**
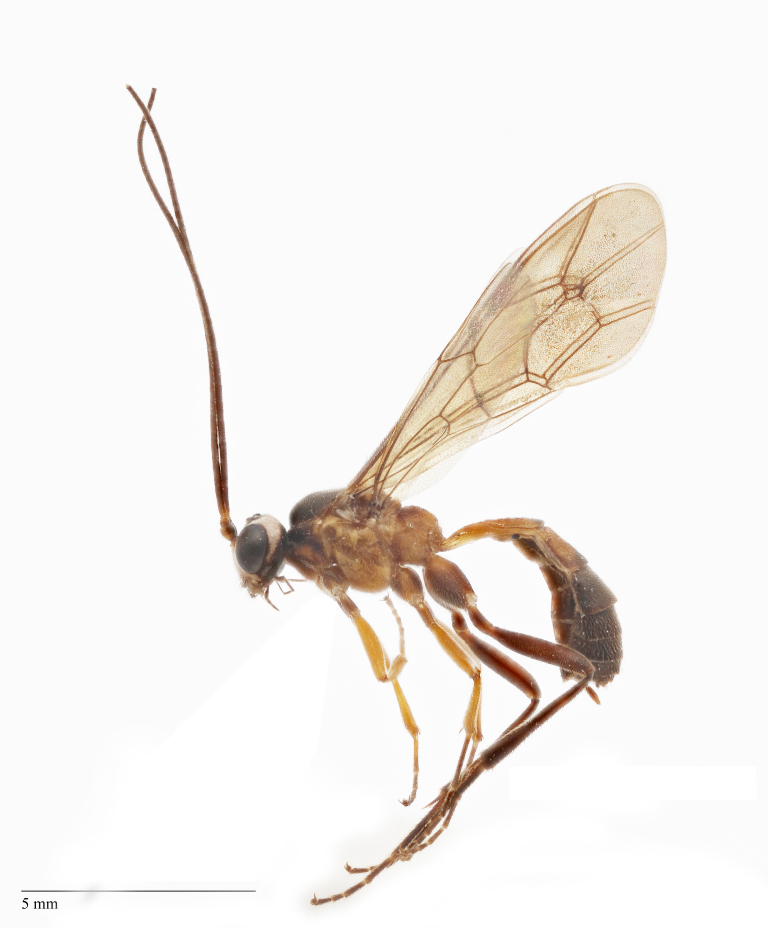
habitus;

**Figure 1b.**
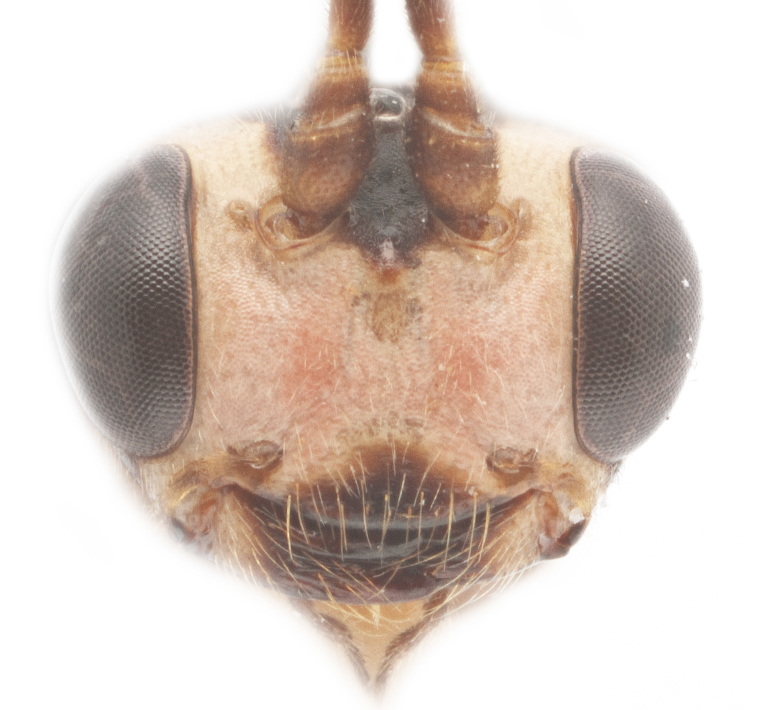
face.

**Figure 2a.**
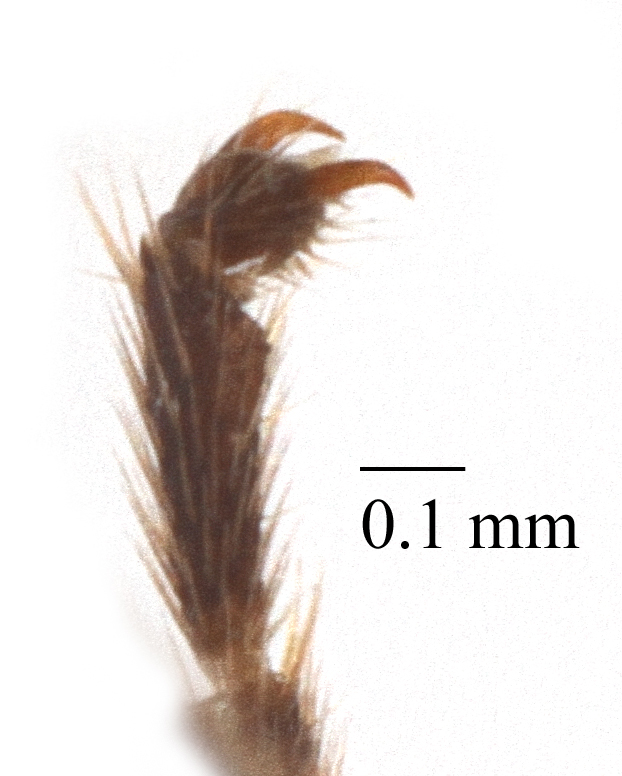
claw;

**Figure 2b.**
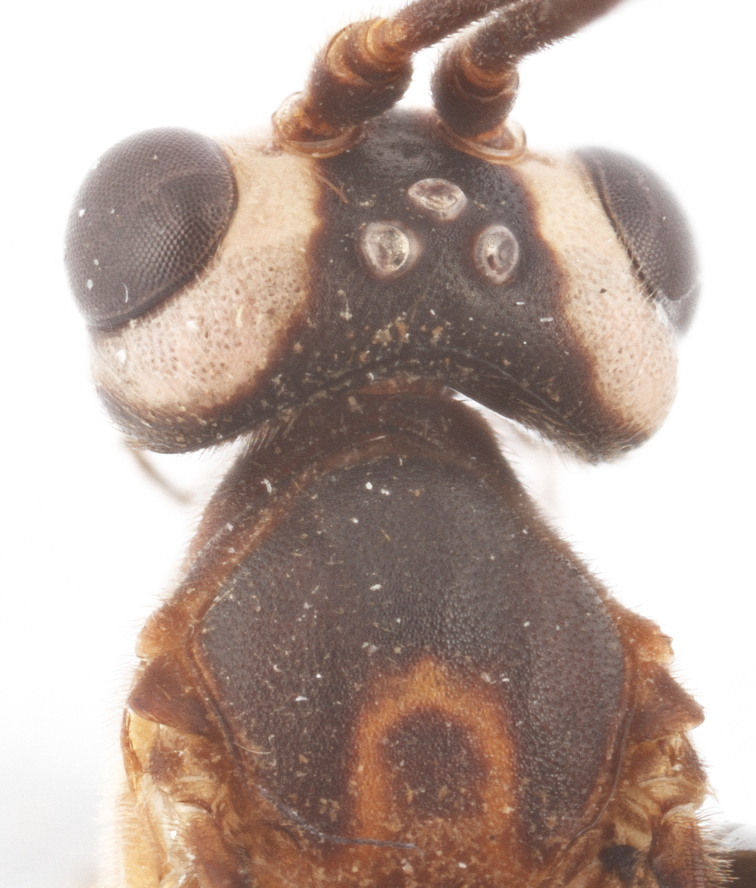
head;

**Figure 2c.**
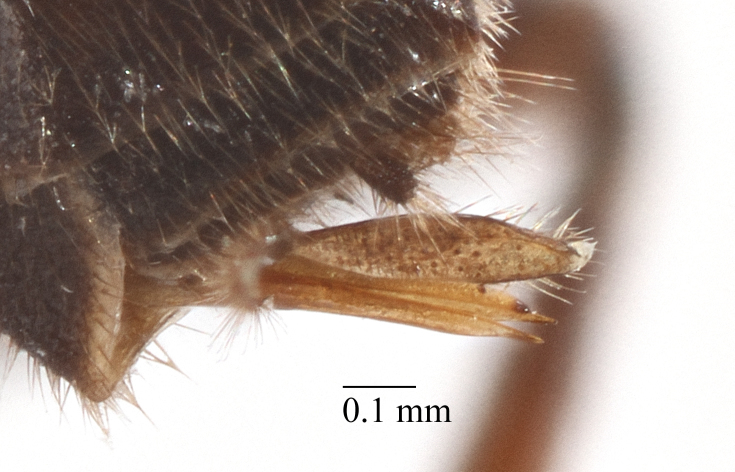
ovipositor;

**Figure 2d.**
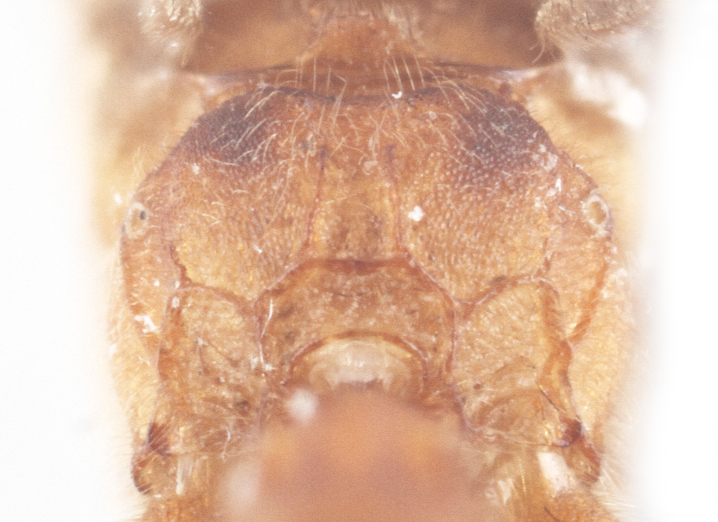
propodeum;

**Figure 2e.**
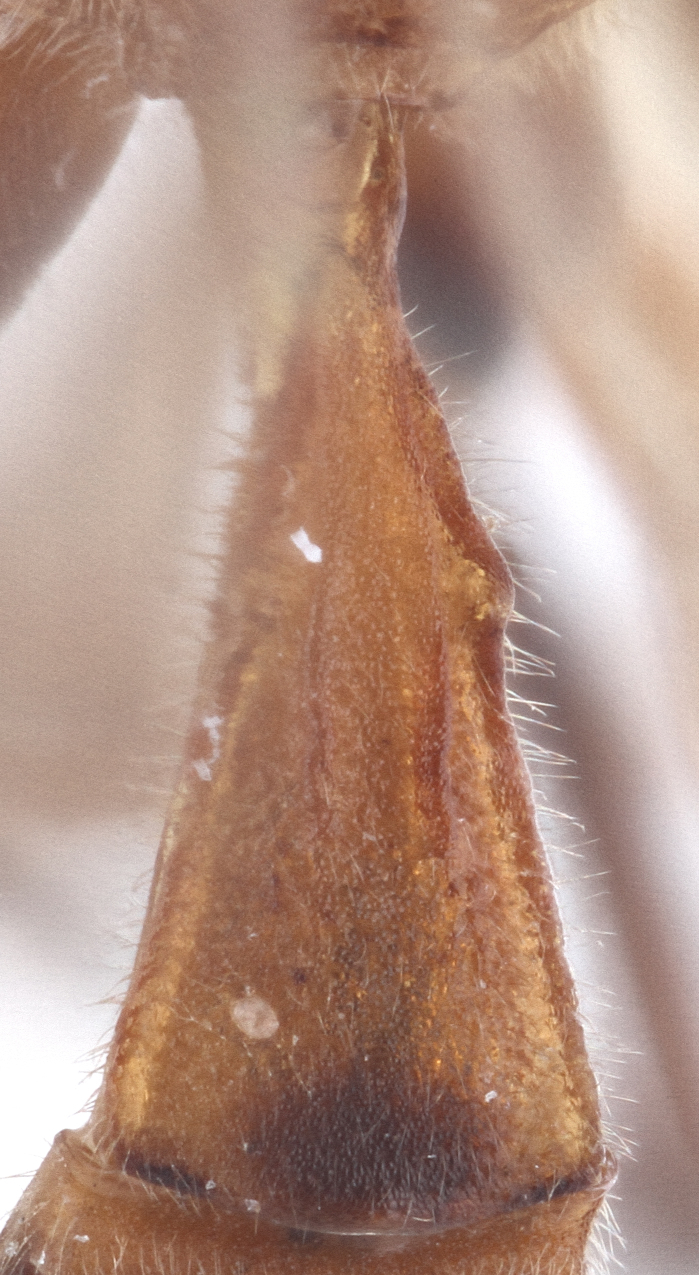
1st metasomal tergite;

**Figure 2f.**
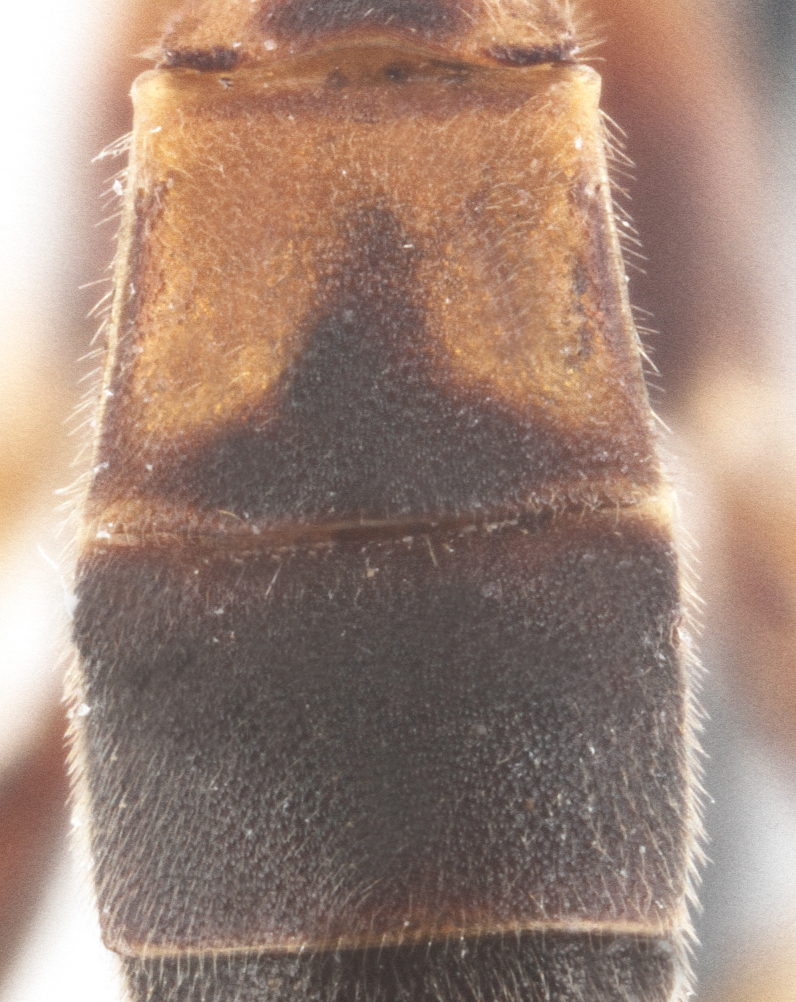
2nd metasomal tergite.

**Figure 4a.**
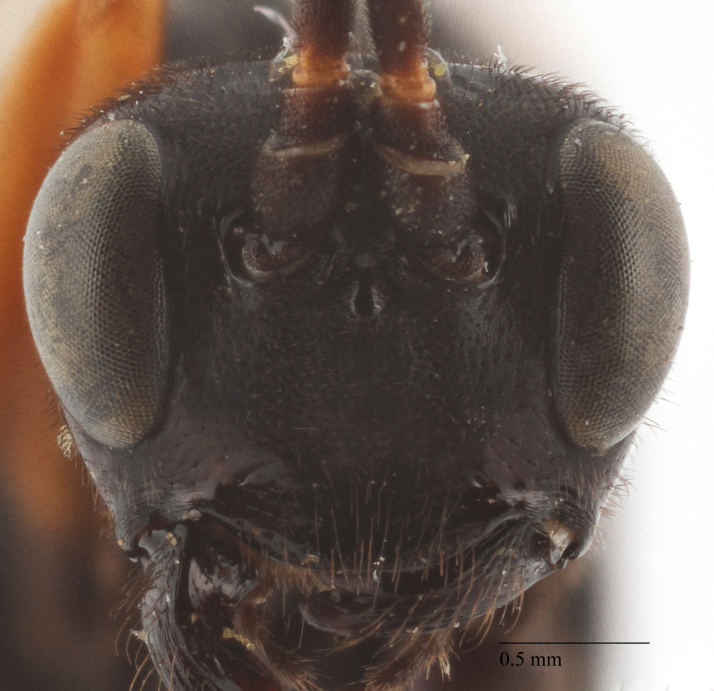
face;

**Figure 4b.**
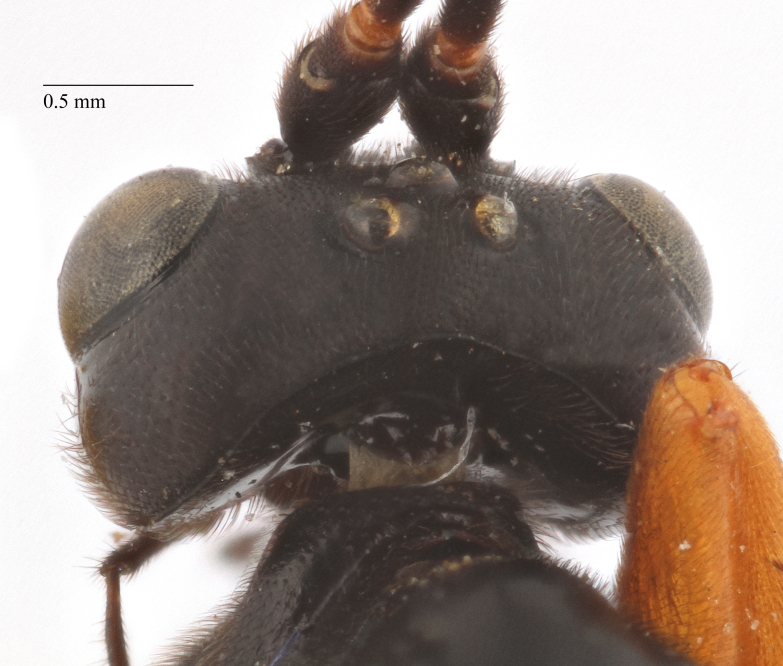
head;

**Figure 4c.**
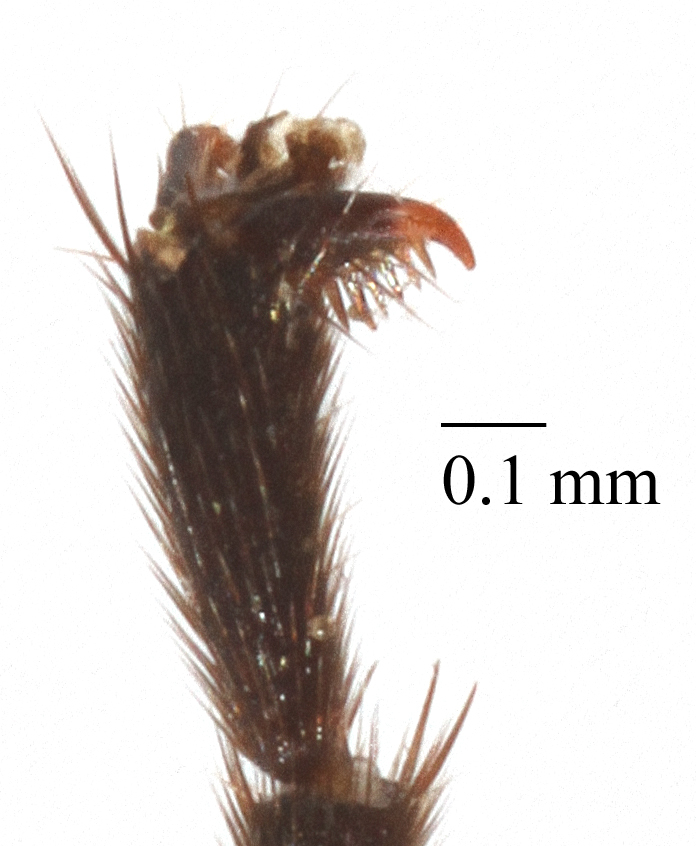
claw;

**Figure 4d.**
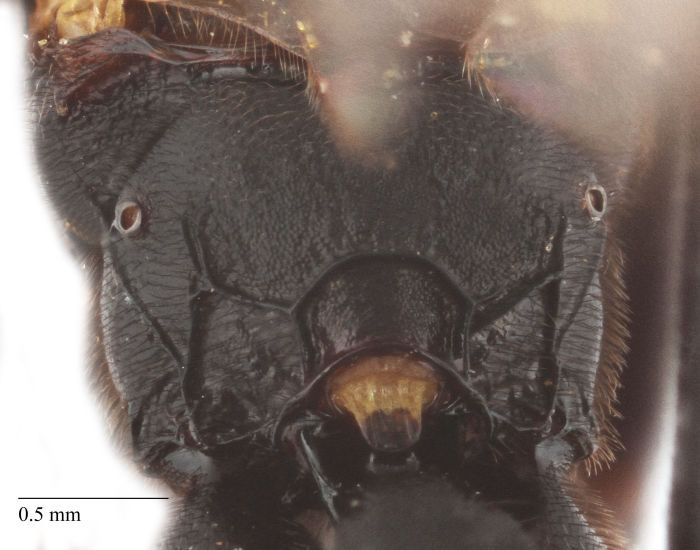
propodeum;

**Figure 4e.**
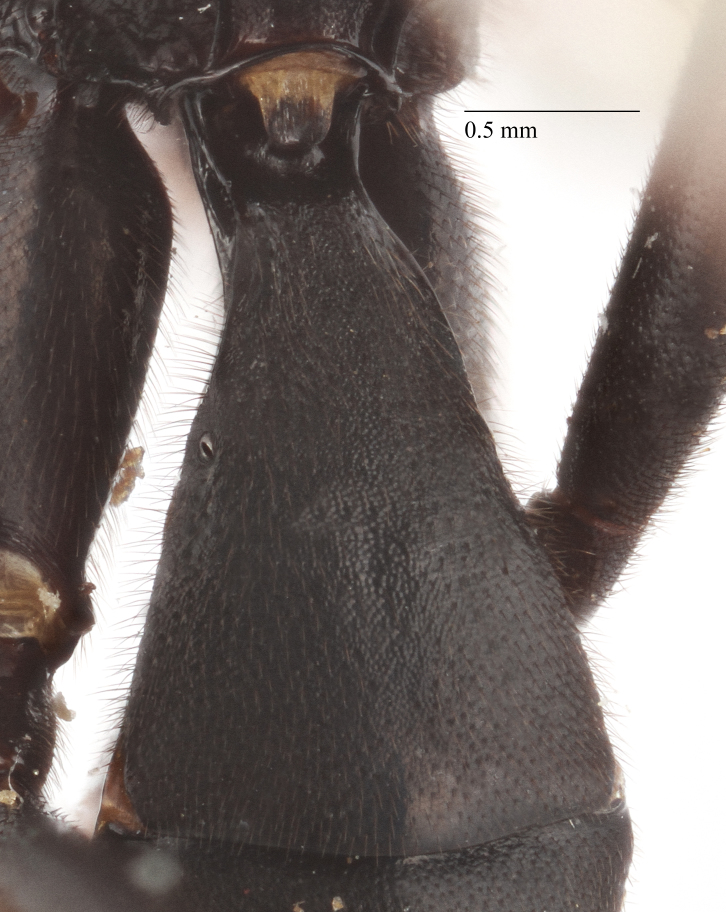
1st metasomal tergite;

**Figure 4f.**
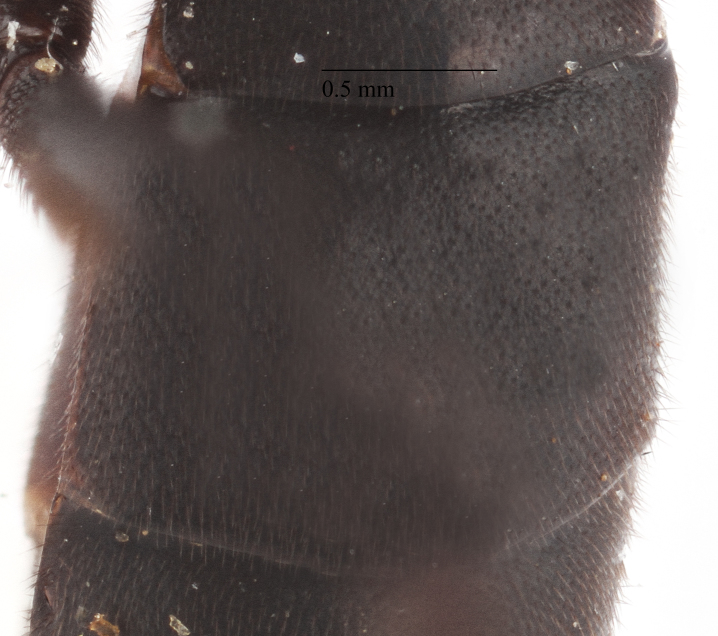
2nd metasomal tergite.
